# Quantification of the nonlinear susceptibility of the hydrogen and deuterium stretch vibration for biomolecules in coherent Raman micro‐spectroscopy

**DOI:** 10.1002/jrs.6164

**Published:** 2021-06-23

**Authors:** Dale Boorman, Iestyn Pope, Francesco Masia, Peter Watson, Paola Borri, Wolfgang Langbein

**Affiliations:** ^1^ School of Biosciences Cardiff University Cardiff UK; ^2^ School of Physics and Astronomy Cardiff University Cardiff UK

**Keywords:** CARS, coherent Raman, deuterium, isotopes, Raman

## Abstract

Deuterium labelling is increasingly used in coherent Raman imaging of complex systems, such as biological cells and tissues, to improve chemical specificity. Nevertheless, quantitative coherent Raman susceptibility spectra for deuterated compounds have not been previously reported. Interestingly, it is expected theoretically that –D stretch vibrations have a Raman susceptibility lower than –H stretch vibrations, with the area of their imaginary part scaling with their wavenumber, which is shifted from around 2900 cm^−1^ for C–H into the silent region around 2100 cm^−1^ for C–D. Here, we report quantitative measurements of the nonlinear susceptibility of water, succinic acid, oleic acid, linoleic acid and deuterated isoforms. We show that the –D stretch vibration has indeed a lower area, consistent with the frequency reduction due to the doubling of atomic mass from hydrogen to deuterium. This finding elucidates an important trade‐off between chemical specificity and signal strength in the adoption of deuterium labelling as an imaging strategy for coherent Raman microscopy.

## Graphical abstract

Deuterium labelling is an important tool in coherent Raman imaging. Quantitative coherent Raman susceptibility spectra of water, succinic acid, oleic acid, linoleic acid and deuterated isoforms, separated into real and imaginary part, are reported. The theoretical prediction that –D stretch vibrations have a Raman susceptibility lower than –H stretch vibrations according to their reduced frequency is verified and quantified.

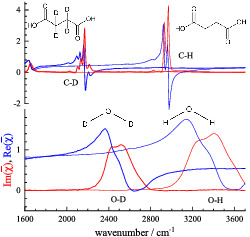

## 1 INTRODUCTION

Raman micro‐spectroscopy is increasingly used as a label‐free, chemically specific imaging modality in cell biology.^[^ ^1^ ^]^ Spontaneous Raman scattering cross‐sections of biomolecules, however, are notoriously small, with corresponding limitations in imaging speed and sensitivity. The development of coherent Raman scattering (CRS) microscopy^[^ ^2^ ^]^ is partially circumventing these limitations, owing to the constructive interference of Raman scattering by identical bonds in the focal volume, coherently driven to vibrate in phase. Many of the earlier demonstrations of CRS microscopy^[^ ^3^, ^4^ ^]^ showed images acquired at a single vibrational resonance, often chosen at the CH_2_ symmetric stretch vibration around 2850 cm^−1^ wavenumber, which is abundant in lipids. It was soon realized, however, that this approach offered limited chemical specificity, considering that many biomolecules have C–H bonds and that the C–H stretch region is congested with several overlapping resonances. Substituting hydrogen with deuterium offers a simple way to increase chemical specificity, considering that C–D stretch vibrations are shifted into the so‐called silent region of the vibrational spectrum, around 2100 cm^−1^, so that they can be easily distinguished. Indeed, deuterated compounds have been used extensively to provide specific chemical contrast when examining lipid bilayers,^[^ ^5^, ^6^ ^]^ drug delivery,^[^ ^7^ ^]^ protein synthesis,^[^ ^8^ ^]^ metabolic dynamics,^[^ ^9^ ^]^ glucose metabolism,^[^ ^10^ ^]^ and neurodegenerative disorders.^[^ ^11^ ^]^

Triggered by these developments, a basic question arises, namely, how is the magnitude of the Raman scattering cross‐section changing upon deuterium substitution, and in turn, how is the CRS susceptibility *amplitude* scaling. In other words, besides introducing a vibrational resonance frequency shift, what are the implications of deuterium labelling on the Raman and CRS signal strength? Surprisingly, despite the fundamental nature of the question, there have been only few reports on the comparison of absolute Raman scattering cross‐sections of molecules as function of their deuteration, and only by means of spontaneous Raman scattering, while a quantitative comparison of the CRS susceptibility of deuterated versus nondeuterated compounds is still missing.

In this work, we have quantitatively measured the nonlinear susceptibility strength for a series of deuterated compounds and compared the results with theoretical expectations derived from spontaneous and coherent Raman polarizability models. Measurements were carried out with our home‐built hyperspectral coherent anti‐Stokes Raman scattering (CARS) microscope and an in‐house developed unsupervised quantitative data‐analysis algorithm, which allows us to retrieve Raman‐like spectra and spatially resolved concentration maps of chemical components, in physically meaningful units, with no prior knowledge.^[^ ^12^, ^13^, ^14^, ^15^ ^]^ With this method, we can determine the CRS susceptibility $\bar{\chi }$ in absolute units, by referencing it to the one of standard microscopy glass^[^ ^16^ ^]^ which has a known nonlinear susceptibility. We have used the method to measure $\bar{\chi }$ of deuterated and nondeuterated isoforms of selected biological compounds, namely, water, succinic acid, oleic acid and linoleic acid. Additionally, the relative spontaneous Raman scattering intensities were measured and analysed.

The paper is organized as follows. In Section 2, we introduce the effect of changing isotopes for the Raman response, as predicted by the bond polarizability model. In Section 3, we provide details of materials and methods, and in Section 4, we report the measured Raman and $\bar{\chi }$ spectra for the compounds studied.

## 2 ISOTOPE EFFECTS IN RAMAN SUSCEPTIBILITIES

### 2.1 Spontaneous Raman scattering

In the bond polarizability model, the differential Raman cross‐section of a vibrational mode *l* is given by^[^ ^17^ ^]^ (see supporting information, Section S1)

(1) $$\frac{d\sigma_{l}}{d\Omega }=\frac{\mu_{0}^{2}}{1440\pi^{2}}\frac{{\left(\omega_{0}-\omega_{l}\right)}^{4}}{1-\exp(-\hbar \omega_{l}/k_{B}T)}S_{l},$$

where *μ*_0_ is the vacuum permeability, *ω*_0_ is the incoming light angular frequency, *ω*_*l*_ is the angular frequency of the vibrational mode, *k*_B_ is the Boltzmann constant and *T* the temperature. *S*_*l*_ is the scattering coefficient with can be expressed for unpolarized detection in a direction orthogonal to the excitation polarization as

(2) $$S_{l}=45{\left({{\bar{\alpha }}_{l}}^{\prime }\right)}^{2}+7{\left({{\bar{\gamma }}_{l}}^{\prime }\right)}^{2},$$

where ${\left({{\bar{\alpha }}_{l}}^{\prime }\right)}^{2}$ and ${\left({{\bar{\gamma }}_{l}}^{\prime }\right)}^{2}$ are the invariants^[^ ^18^ ^]^ of the derivative of the molecular polarizability tensor with respect to the dimensionless normal coordinate *q*_*l*_. Here, $45{\left({{\bar{\alpha }}_{l}}^{\prime }\right)}^{2}$ provides the isotropic (or trace) part, which is co‐polarized to the excitation polarization, and $7{\left({{\bar{\gamma }}_{l}}^{\prime }\right)}^{2}$ is the anisotropic part, of which $4{\left({{\bar{\gamma }}_{l}}^{\prime }\right)}^{2}$ is co‐polarized and $3{\left({{\bar{\gamma }}_{l}}^{\prime }\right)}^{2}$ is cross‐polarized. *q*_*l*_ is related to the coordinates *x*_*i*_ of the atom *i* with mass *m*_*i*_ in the molecule by

(3) $$x_{i}=\underset{l}{\sum }q_{l}D_{li}\sqrt{\frac{\hbar }{\omega_{l}m_{i}}}$$

with the orthonormal transformation matrix *D*_*ij*_ describing the eigenmodes. Changing the mass of an atom by replacing it with an isotope preserves the electronic binding and thus the force constants *K*_*ij*_ of the vibrational dynamics. This leads to an approximate sum rule^[^ ^19^ ^]^

(4) $$\underset{l}{\sum }\frac{S_{l}}{\omega_{l}}=s$$

with a constant *s* independent of the isotopes. While the sum extends over all vibrations of the molecule, due to the much smaller mass of both hydrogen and deuterium compared with other atoms, their stretching vibrations are often not significantly mixing with others. Hence, one can expect that the sum rule holds for the –H and –D stretch vibrations alone, predicting that the scattering coefficient scales proportional to the vibrational frequency. We note that equivalent sum rules hold also for the infrared polarizability of the vibrational modes.^[^ ^19^, ^20^ ^]^

A similar conclusion can be reached considering an isolated stretch vibration and an infinite mass of the C atom. In this case, we can use $\omega_{l}^{2}=K_{ll}/m_{l}$, so that $x_{l}=q_{l}\sqrt{\hbar \omega_{l}/K_{ll}}$, and it follows that

(5) $${\left({\bar{\alpha }}_{l}^{\prime }\right)}^{2}={\left(\frac{\partial \bar{\alpha }}{\partial q_{l}}\right)}^{2}={\left(\frac{\partial \bar{\alpha }}{\partial x_{l}}\right)}^{2}\frac{\hbar \omega_{l}}{K_{ll}}\;,$$

and equivalently for ${\left({{\bar{\gamma }}_{l}}^{\prime }\right)}^{2}$. Because the derivative of the polarizabilities versus *x*_*l*_ is a property of the electronic binding and therefore independent of the isotope mass, we find that the scattering coefficient is proportional to *ω*_*l*_.

**FIGURE 1:**
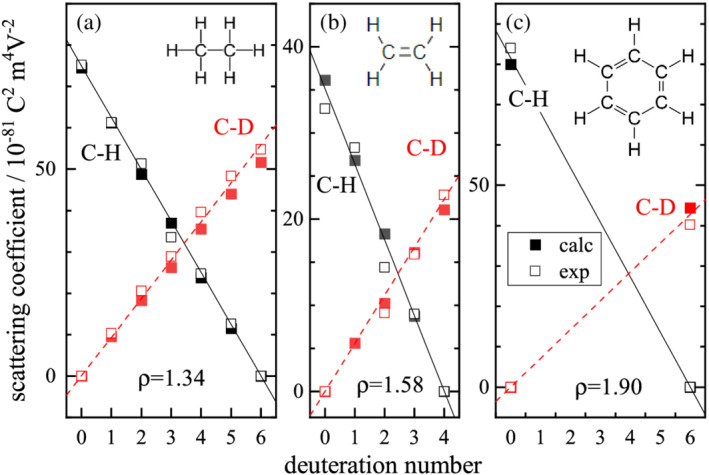
Raman scattering coefficients of the C–H stretch vibrations (around 3000 cm^−1^) and corresponding C–D stretch vibrations (around 2200 cm^−1^) for ethane^[^ ^17^ ^]^ (a), ethene^[^ ^21^ ^]^ (b) and benzene^[^ ^22^ ^]^ (c), versus deuteration number. The open symbols are measured data; the closed symbols are results of a fit by a bond‐polarizability model. Lines are fits proportional to bond number. The ratio *ϱ* between the scattering coefficient per bond for C–H relative to C–D corresponding to the fits is given

There have been only few experimental reports on the comparison of absolute Raman scattering cross‐sections of molecules as function of their deuteration, and none directly addressed the theoretical prediction of a scattering coefficient scaling proportionally to the vibrational frequency. Let us summarize the experimental finding of these works in relation to the sum rule given by Equation (4). In Martín and Montero,^[^ ^17^ ^]^ ethane was measured in the gas phase and fitted with a bond‐polarizability model, followed by ethene^[^ ^21^ ^]^ and benzene.^[^ ^22^ ^]^ The reported scattering cross‐sections for C–H and C–D stretch vibrations are used here to evaluate the sum rule prediction. The sum of the reported scattering coefficients *S*_*l*_ of the C–H stretching vibrations *S*_H_ (2800–3150 cm^−1^) and the C–D stretching vibrations *S*_D_ (2100–2400 cm^−1^) are shown in Figure 1 versus the deuteration number. The scattering coefficients are well described by a proportionality with the number of C–H and C–D bonds, respectively, yielding values of (*S*_H_, *S*_D_) per bond in units of 10^−81^ C^2^m^4^V^−2^ given by (12.8, 9.3) for ethane, (8.8, 5.6) for ethene, and (13.6, 7.1) for benzene. The corresponding ratio $\varrho =S_{H}/S_{D}$ is 1.34 for ethane, 1.58 for ethene and 1.9 for benzene. Absolute measurement errors are estimated to be around 10% in these works.^[^ ^17^, ^21^, ^22^ ^]^ The largest systematic uncertainty of the ratio is present in benzene where no partially deuterated data were reported. Let us now compare *ϱ* with the vibrational wavenumber ratios. For ethane, the average C–H stretch vibrations of C_2_H_6_ are at 2942 cm^−1^, and the average C–D stretch vibrations of C_2_D_6_ are 2152 cm^−1^, having a wavenumber ratio of 1.37. This is consistent with the measured *ϱ*. For ethene, the averages are 3051 cm^−1^ for of C_2_H_4_ and 2277 cm^−1^ for C_2_D_4_, yielding a ratio of 1.34, which is lower than the measured *ϱ* of 1.58. For benzene, the averages are 3068 and 2291 cm^−1^, yielding a ratio of 1.34, which is significantly lower than the measured *ϱ* of 1.9. Evaluating the sum rule Equation (4) over all vibrations reported in other studies,^[^ ^17^, ^21^, ^22^ ^]^ we find that the constant *s* changes by a factor 1.09 from C_2_H_6_ to C_2_D_6_, 0.824 from C_2_H_4_ to C_2_D_4_, and 1.16 from C_6_H_6_ to C_6_D_6_. The deviation from 1 (i.e., a constant *s* independent of the isotopes) is close to the reported measurement error; hence, we can conclude that the scaling of the scattering coefficients with the vibrational frequency for deuteration is approximately valid for these examples. Notably, considering the reduced masses of the C–D bond μ=Da/(1/2+1/12), and the C–H bond μ=Da/(1+1/12) and the scaling of the frequency with the reduced mass as $1/\sqrt{\mu }$, we expect a frequency ratio of 1.363, very close to the observed value for all molecules, confirming that the force constants *K*_*ll*_ are not significantly changed by deuteration.

**FIGURE 2:**
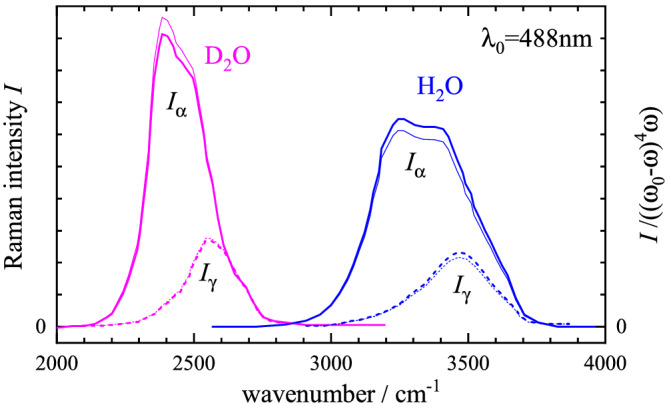
Raman scattering intensities^[^ ^23^ ^]^ of water (H_2_O, blue) and heavy water (D_2_O, magenta) at 23°C using 488‐nm excitation. The isotropic part *I*_*α*_ is shown as solid line, and the anisotropic part *I*_*γ*_ as dashed line. The intensities scaled by a factor (*ω*_0_ − *ω*)^−4^ *ω* ^−1^ are shown as thin lines

For liquids, the effect of deuteration has been studied in water. Calibrated Raman scattering intensities are given in fig. 8 of Scherer et al.,^[^ ^23^ ^]^ on the same scale for water (H_2_O) and heavy water (D_2_O), and are shown in Figure 2. The intensities were measured co‐polarized (*I*_||_) and cross‐polarized (*I*_⊥_) to the linear excitation polarization and are separated into the isotropic part $I_{\alpha }=I_{\left|\right|}-I_{\gamma }$, shown as solid line, and the anisotropic part $I_{\gamma }=\frac{4}{3}I_{⊥}$, shown as dashed line. *I*_*α*_ corresponds to the scattering coefficient $S_{l}=45{\left({{\bar{\alpha }}_{l}}^{\prime }\right)}^{2}$ and *I*_*γ*_ to $4{\left({{\bar{\gamma }}_{l}}^{\prime }\right)}^{2}$ in Equation (1). Integrating the Raman intensities over the bands shown in Figure 2  and scaling the integrated intensities with (*ω*_0_ − *ω*_*l*_)^−4^ (the thermal occupation $\exp(-\hbar \omega_{l}/k_{B}T)$ is less than 10^−4^ at room temperature for wavenumbers above 2000 cm^−1^ and thus can be neglected for C–D and C–H stretch vibrations), where we have assigned a centre wavenumber of 3300 cm^−1^ for H_2_O and 2450 cm^−1^ for D_2_O, we find a ratio of ϱ=1.44 for *I*_*α*_ and 1.42 for *I*_*γ*_, while the wavenumber ratio is 1.35. This is in reasonable agreement with the expected scaling. Because the Raman spectra are rather broad, we have also evaluated the sum rule directly, using the integrals of the intensities scaled by *ω* ^−1^(*ω*_0_ − *ω*)^−4^ (see thin lines in Figure 2), yielding the ratio of 1.07 for *I*_*α*_ and 1.06 for *I*_*γ*_ and thus showing a deviation from the sum rule below 10%.

The Raman cross‐sections H_2_O and D_2_O in gaseous form are reported in Avila et al.^[^ ^24^ ^]^ tab. 3, for an excitation wavelength of 514.5 nm at T=300 K. Using the centre frequencies of the dominating Q branch of *ν*_1_ at 3650 and 2670 cm^−1^, we find a ratio ϱ=1.44 for *I*_||_ and 1.45 for *I*_⊥_, with a wavenumber ratio of 1.37, similar to liquid water.

### 2.2 Coherent Raman scattering

CRS uses two incident light fields, called pump and Stokes, to coherently drive molecular vibrations at the frequency difference between these two fields. The coherent Raman susceptibility for a simple harmonic oscillator of frequency *ω*_*l*_, mass *m*_*l*_, is given by^[^ ^25^ ^]^

(6) $$\chi_{R}=\frac{\epsilon_{0}N}{6m_{l}}{\left(\frac{\partial \alpha }{\partial x_{l}}\right)}^{2}{\left(\omega_{l}^{2}-\Delta^{2}+2i\Delta \gamma \right)}^{-1},$$

where $\Delta =\omega_{P}-\omega_{S}$ is the difference between the pump and Stokes angular frequency, *N* is the density of oscillators and *∂α/∂x*_*l*_ is the derivative of the polarizability versus the position coordinate. Close to resonance, |Δ/*ω*_*l*_ ± 1|≪1, we can approximate *χ*_R_ as (see supporting information, Section S2)

(7) $$\chi_{R}\approx \frac{\epsilon_{0}N}{12m_{l}\omega_{l}}{\left(\frac{\partial \alpha }{\partial x_{l}}\right)}^{2}{\left(\omega_{l}\mp \Delta \pm i\gamma \right)}^{-1}\;,$$

having an imaginary part of

(8) Im(χR)≈ϵ0N12mlωl∂α∂xl2∓γ(ωl∓Δ)2+γ2.

The area of ℑ(*χ*_R_) over the resonance is given by

(9) A=∫Im(χR)dΔ≈∓πϵ0N12mlωl∂α∂xl2.

Now, for isotope substitution, we can again use $\omega_{l}^{2}=K_{ll}/m_{l}$, so that

(10) $$A\approx \mp \frac{\pi \epsilon_{0}N\omega_{l}}{12K_{ll}}{\left(\frac{\partial \alpha }{\partial x_{l}}\right)}^{2}\;.$$

We therefore find that the area *A* is proportional to the vibrational angular frequency *ω*_*l*_, hence follows the same scaling as the Raman scattering coefficient discussed in Section 2.1.

In terms of available experimental findings, a previous Raman and CARS study of deuterated compounds^[^ ^26^ ^]^ compared the measured spontaneous Raman intensity of the C–D band to the intensity of the dimethyl sulfoxide (DMSO) solvent, and also showed a ratiometric CARS intensity analysis of a deuterated versus nondeuterated compound. The latter study however was carried out without retrieval of the CRS susceptibility, which for CARS intensities leads to a complicated response affected by the nonresonant background. We are not aware of a quantitative comparison of the CRS susceptibility of deuterated versus nondeuterated compounds.

## 3 MATERIALS AND METHODS

### 3.1 Materials

Pure (>99%) succinic acid (SA), a dicarboxylic acid and D4‐succinic acid (D4‐SA), with four deuterium atoms bound to carbons 2 and 3, were purchased from Sigma Aldrich (UK). Pure (>99%) oleic acid (OA—a monosaturated 18 C molecule with a *cis* double bond at the ninth carbon), D17‐oleic acid (D17‐OA—having 17 deuterium atoms attached to carbons 11–18), linoleic acid (LA—a biunsaturated 18 C molecule with two *cis* double bonds at the ninth and twelfth carbons) and D11‐linoleic acid (D11‐LA—having 11 deuterium atoms attached to carbons 14–18) were purchased from Cayman Chemical (MI, USA). Heavy water (deuterium oxide, 99.9% deuterated) was purchased from Sigma Aldrich (UK), and distilled water was purchased from Fisher Scientific (UK)—the structures of each of the investigated molecules are provided in Table S1.

### 3.2 Sample preparation

**FIGURE 3:**
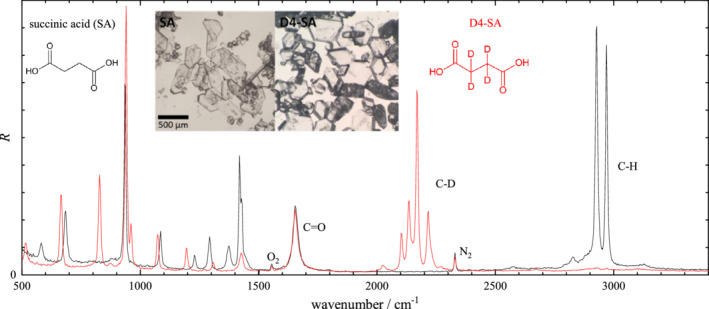
Scaled Raman scattering intensity *R* from pure SA and D4‐SA crystals. Spectra are normalized to the area of their C=O peak at 1660 cm^−1^. The peak area over the C–H range (2700–3200 cm^−1^) is 4.35 for SA, and 0.14 for SA‐D4 due to the O–H vibrations, while over the C–D range (1950–2450 cm^−1^) the peak area is 3.39 for SA‐D4 (not including the N_2_ peak area). The inset shows representative transmission images of the investigated SA and D4‐SA crystals

Crystals of pure SA and D4‐SA were formed by preparing a saturated solution of the compound dissolved in water at a temperature of 90°C and continuous mixing. After cooling to room temperature, 30 *μ*l of the supersaturated solution was transferred into a well of 9‐mm diameter, and 120‐*μ*m thickness formed using an adhesive imaging gasket (Grace BioLabs, OR, USA) attached on a 1‐mm thick, 76 ×26 mm glass microscope slide (Thermo Scientific Menzel Gläser, UK). Samples were stored under humidified conditions to permit slow evaporation over 5 days, allowing large (about 500 *μ*m sized) crystals to form (see inset in Figure 3). Crystals were enclosed with a glass coverslip (thickness #1.5, 25‐mm diameter, PA, VWR International, USA) prior to imaging. OA, D17‐OA, LA and D11‐LA samples were prepared by transferring 10 *μ*m of lipid solution (as provided by manufacturer; OA and LA in ethanol at 100 mg/ml, D17‐OA and D11‐LA in methyl acetate at 100 mg/ml) into the well formed by an imaging gasket attached on a microscope slide, and then evaporating the solvent under gentle nitrogen stream to leave only pure lipid. Consecutive 10 *μ*l aliquots of solution were added and the solvent evaporated until a sufficiently large droplet of pure lipid was generated. The droplets were enclosed with a glass coverslip prior to imaging. Water and heavy water samples were formed by transferring 13 *μ*l of liquid into the centre of an imaging gasket on a glass microscope slide.

### 3.3 CARS

CARS data were acquired using a custom‐built multimodal laser scanning CARS microscope, the layout of which has been described in detail in our previous works.^[^ ^15^, ^27^, ^28^ ^]^ In short, a Ti:Sa broadband (660 to 970 nm) pulsed laser (Venteon, Pulse:One PE) was pumped by a Nd:vanadate laser (Laser Quantum, Finesse) at 4.5 W to generate <8 fs pulses at a repetition rate of 80 MHz and with an average output power of 500 mW. A dichroic beamsplitter (CVI Melles Griot, LWP‐450RP670‐TP830‐PW‐1025‐C) was used to separate (and then recombine) the laser beam into the pump and Stokes components, centred at 682 and 806 nm with a bandwidth (at 10% of the maximum) of 65 and 200 nm, respectively. Spectral focusing through linear chirping of pump and Stokes beams by glass blocks produced pulses of 1.5‐ and 5‐ps duration, respectively, achieving a vibrational spectral resolution of 10 cm^−1^. CARS spectra could be acquired over the range 1200–3800 cm^−1^  without adjustments to the laser, by automated control of the relative delay time between pump and Stokes pulses, and automated switching of detection filters and Stokes glass block thickness. Following recombination, the excitation beams were coupled into a commercial microscope stand (Nikon Ti‐U) via a custom‐built scanning head. Different objective and condenser combinations were used: a 60× 1.27NA water immersion objective (Nikon CFI Plan Apochromat IR *λ*S, MRD70650) with a 1.34NA oil condenser (Nikon T‐C‐HNAO, MEL41410), as well as a 40× 1.15NA water immersion objective (Nikon CFI Plan Apochromat *λ*S, MRD77410) or a 20× 0.75NA dry objective (Nikon CFI Plan Apochromat *λ*, MRD00205) with a 0.72NA dry condenser (Nikon T‐C‐CLWD, MEL56100). CARS signal from samples on microscope slides was collected in the forward direction by a photomultiplier tube (PMT) (Hamamatsu H7422‐40). Use of double Semrock FF01‐609/57, FF01‐593/40 or FF01‐562/40 bandpass filters permitted CARS imaging within the fingerprint (1200–1800 cm^−1^), silent (1800–2600 cm^−1^) and CH‐stretch (2600–3700 cm^−1^) regions of the Raman spectrum, respectively. Nonresonant CARS spectra were acquired from the glass coverslip under the same excitation and detection conditions and used for normalization to correct for the varying temporal overlap of pump and Stokes and to derive a CARS intensity ratio independent of excitation/detection parameters. Both *xy* sample motion and objective motion in *z* direction were motorized (Prior, ProScan III controller (V31XYZ), *xy* stage (H117NN2N) and *z* drive (PS3H122)).

Hyperspectral CARS was acquired using a pixel dwell time of 1 ms, and a PMT gain of 10^5^. Typical excitation powers at the sample were 29 mW for the pump and 21 mW for the Stokes beam. Line scans of a few 10 *μ*m width, having a few hundred pixels, were acquired over the nominal range 1600–4400 cm^−1^, with 5‐cm^−1^ step size. This range includes regions without temporal overlap, which were used for background subtraction.

The reference spectra in glass were taken using the same settings, moving the focus 40 *μ*m into the coverslip glass.

A detailed description of the CARS data processing can be found in our previous works.^[^ ^12^, ^16^, ^29^ ^]^ CARS intensity ratios were calculated by dividing background‐corrected CARS intensities obtained from the investigated bulk materials (crystals or liquids) by the corresponding nonresonant CARS intensity measured in glass under the same excitation and detection conditions. The phase‐corrected Kramers–Kronig method (PCKK) was then used to retrieve from the CARS intensity ratio spectra, $|\bar{\chi }{|}^{2}$, the complex CARS third‐order susceptibility spectra, $\bar{\chi }$, normalized to the nonlinear susceptibility^[^ ^30^ ^]^ $\chi_{1111}^{\left(3\right)}=n_{0}n_{2}/(12\pi )$ of the glass used, with refractive index *n*_0_ and nonlinear refractive index *n*_2_. Considering the Schott D263 glass used for the coverslips in our study, we calculate $n_{2}=1.38\times 10^{-13}$ esu using eq. 39 in Boling^[^ ^31^ ^]^ with $\nu_{d}=54.5$ and $n_{d}=1.5231$. The units of $\bar{\chi }$ presented in this paper are accordingly 5.6 × 10^−15^ esu. The spectral calibration of the spectral focusing, providing the wavenumber calibration in the CARS data, has systematic errors similar to its spectral resolution.

### 3.4 Spontaneous Raman scattering

Spontaneous Raman scattering was acquired using the same microscope as used for CARS described above. A 40× 0.95NA dry objective (Nikon CFI Plan Apochromat *λ*S Series, MRD00405) was used for excitation and collection. The excitation beam from a 532‐nm laser (Laser Quantum GEM) was passed through an exciter filter (Semrock LL01‐532) and coupled into the microscope stand left port via a dichroic mirror (Semrock LPD01‐532RS). Raman scattering was collected in epi‐direction, transmitted through the dichroic and a long pass filter (Semrock BLP01‐532R), dispersed by a spectrometer (Horiba Jobin‐Yvon iHR 550) with a grating of 300 lines per millimetre blazed at 600 nm, and detected by a charge‐coupled device (CCD) camera (Andor Newton DU971N‐BV cooled to −60°C). Imaging of the intermediate image plane at the left port of the Ti‐U microscope stand onto a horizontal slit (Thorlabs VA100) and then onto the vertical input slit of the spectrometer provided confocality. The spectral resolution was 13 cm^−1^. All measurements were performed with the samples at room temperature. The data were corrected by the spectral sensitivity, from sample to detection, calibrated using a radiometric calibration source (Ocean Optics LS‐1‐cal‐int‐22) to provide the intensity *I* ^R^(*ω*) proportional to the power per frequency across the spectrum. An exposure time of 20 s and an excitation power of about 100 mW at the sample was used.

## 4 RESULTS

### 4.1 Spontaneous Raman scattering

**FIGURE 4:**
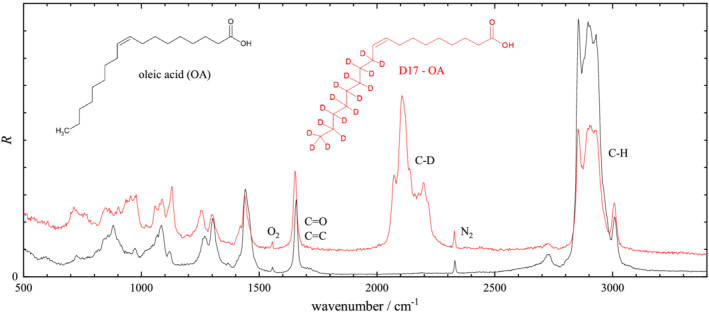
Scaled Raman scattering intensity *R* from oleic acid (OA) and D17‐OA. Spectra are normalized to the area of the (C=O, C=C) peak at 1660 cm^−1^ over the range 1600–1750 cm^−1^. The peak area over the C–H range (2600–3200 cm^−1^) is 18.5 for OA and 9.37 for D17‐OA, while over the C–D range (1950–2450 cm^−1^), the peak area is 9.08 for D17‐OA (not including the N_2_ peak area)

**FIGURE 5:**
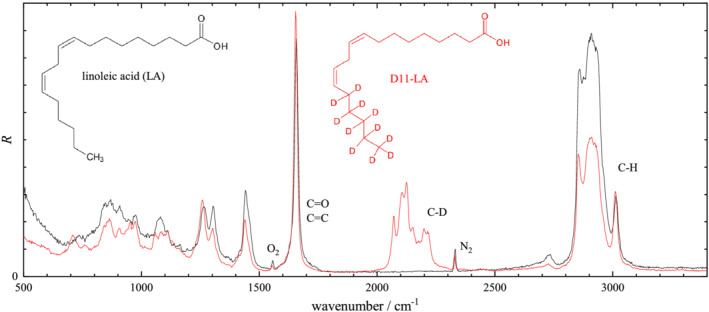
Scaled Raman scattering intensity *R* from linoleic acid (LA) and D11‐LA. Spectra are normalized to the area of the (C=O, C=C) peak at 1660 cm^−1^ over the range 1600–1750 cm^−1^. The peak area over the C–H range (2600–3200 cm^−1^) is 5.27 for LA and 3.13 for D11‐LA, while over the C–D range (1950–2450 cm^−1^), the peak area is 1.56 for D11‐LA (not including the N_2_ peak area)

Spontaneous Raman spectral intensities *I* ^R^ were acquired from the samples as described in Section 3.4. Spectra show a slowly varying background, likely due to fluorescence. This was accounted for in the following analysis using a baseline subtraction for the evaluation of areal integrals around relevant bands. Spectra were converted into the quantity $R=CI^{R}{\left(\omega_{0}-\omega \right)}^{-4}\omega^{-1}$, to be able to directly evaluate the sum rule (4) by spectral integration. The normalization *C* was chosen to have a unity integral of *R* over the C=C and C=O peak at 1660 cm^−1^, evaluated across the range 1600–1750 cm^−1^ using a linear baseline matching the end points. The resulting spectra for both nondeuterated and deuterated isoforms are shown in Figure 3 for succinic acid, Figure 4 for oleic acid and Figure 5 for linoleic acid. The spectra clearly show the C–H and C–D stretch vibrations and a fingerprint region with more complex differences between deuterated and nondeuterated materials. The C–D and C–H peak areas in *R* were evaluated in the 1900–2450 cm^−1^ and 2600–3200 cm^−1^ range, respectively, using a linear baseline matching the end points. The results are given in Table 1, including the peak area per relevant bond. To evaluate the sum rule, the ratio *η* of the area per bond of C–H to C–D is also reported. We see that the determined *η* is in the 1.05 to 1.28 range, confirming the approximate validity of the sum rule for these substances, with deviations in the 10% to 20% range. This is similar to the result from previous reports on other substances given in Section 2.

**TABLE 1:** Areas of the quantity *R*, for spontaneous Raman scattering, corresponding to the C–H and C–D stretch associated peaks, in units of the (C=O, C=C) peak area

Molecule	Bond	*N*_b_	Area	Area/*N*_b_	*η*
Succinic acid	C–H	4	4.35	1.09	1.28
D4‐succinic acid	C–D	4	3.39	0.85	
Oleic acid	C–H	33	18.5	0.561	1.05
D17‐oleic acid	C–H	16	9.37	0.586	1.10
	C–D	17	9.08	0.534
Linolenic acid	C–H	31	5.27	0.170	1.20
D11‐linolenic acid	C–H	20	3.13	0.157	1.11
	C–D	11	1.56	0.142	

*Note*: *N*_b_ is the number of bonds per molecules. The area per bond and the ratio *η* of the area per C–H bond to the area per C–D bond are also given.

Succinic acid (Figure 3) shows the sharpest vibrational lines, many of them resolution limited (13 cm^−1^), consistent with it being in crystalline form. The line arrangement shows that the crystal is in its more stable *β* form.^[^ ^32^ ^]^ The vibrations in the C–H stretch range are the symmetric (2924 cm^−1^) and asymmetric (2967 cm^−1^) stretch.^[^ ^32^, ^33^ ^]^ We are not aware of a detailed line assignment for D4‐SA. It is also interesting to note that because the area per C–H bond is close to unity relative to the C=O area (see Table 1), and considering that in a SA there are two C=O bonds, we can deduce that a C=O bond has about half the area of a C–H bond.

Oleic acid (Figure 4) shows generally broader vibrational lines because it is in liquid form. The C–H stretch region^[^ ^34^ ^]^ shows a sharp 2850 cm^−1^ peak due to the CH_2_ symmetric stretch. The 2720 cm^−1^ band is due a Fermi resonance with the overtones of other CH_2_ vibrations. The =C–H stretch gives rise to a band around 3010 cm^−1^. The line at 1660 cm^−1^ used for normalization contains both the C=O and C=C stretch vibrations. Considering the area per C–H bond (Table 1) and assuming that the absolute scattering coefficients of the C–H bond and C=O bond are equal to those in SA, one can deduce that the C=C stretch has 2×1.09/0.561−1=2.89 times the area of the C=O stretch.

Linoleic acid (Figure 5) is also in liquid form. Compared with OA, the C–H stretch region shows a stronger =C–H stretch at 3010 cm^−1^ due to the presence of four of these bonds, instead of two in OA. The C=C stretch at 1660 cm^−1^ is enhanced even more, with a ratio to the =C–H stretch approximately double than that seen in OA. This indicates a coherent coupling between the two C=C bonds in LA, resulting in a single Raman active mode with twice the polarizability and thus four times the intensity. Considering the area per C–H bond (Table 1) and assuming again the same absolute area of the C–H bond and C=O bond as in SA, one can deduce that the double C=C stretch in LA has 2×1.09/0.170−1=11.8 times the area of the C=O stretch, which is very close to the value of 4×2.89=11.6 resulting from the coherent coupling model. Such coupling, both vibrational and electronic, is the mechanism allowing for large Raman cross‐sections in polyynes,^[^ ^35^ ^]^ which can be used as cellular labels.^[^ ^36^ ^]^

### 4.2 CARS third‐order susceptibility

**FIGURE 6:**
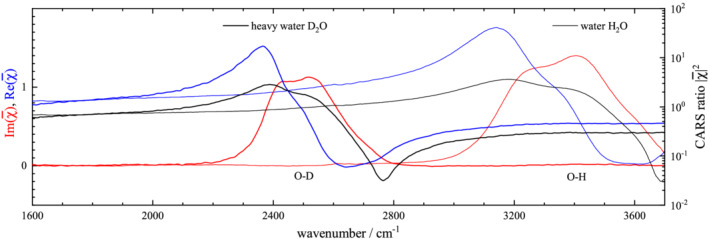
Coherent anti‐Stokes Raman scattering (CARS) ratio $|\bar{\chi }{|}^{2}$ (black), and retrieved real (blue) and imaginary (red) part of the normalized CARS susceptibility $\bar{\chi }$, of water H_2_O (thin lines) and heavy water D_2_O (thick lines), at 20°C

**FIGURE 7:**
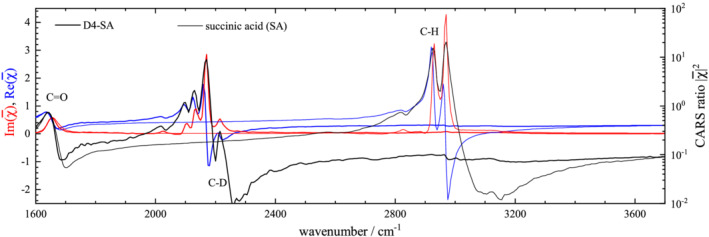
As Figure 6, for SA (thin lines) and D4‐SA (thick lines)

**FIGURE 8:**
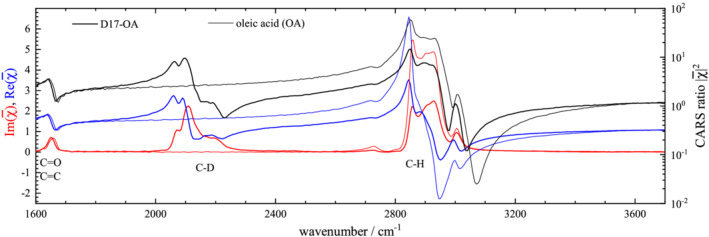
As Figure 6, for OA (thin lines) and D17‐OA (thick lines)

**FIGURE 9:**
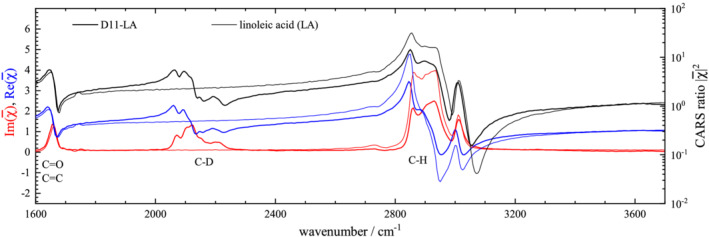
As Figure 6, for LA (thin lines) and D11‐LA (thick lines)

The CARS spectra were acquired on the samples as described in Section 3.3. The measured CARS ratio $|\bar{\chi }{|}^{2}$ and the retrieved complex CARS susceptibility $\bar{\chi }$, in its real and imaginary part, are shown for both nondeuterated and deuterated isoforms, in Figure 6 for water, Figure 7 for succinic acid, Figure 8 for oleic acid and Figure 9 for linoleic acid.

We see that $|\bar{\chi }{|}^{2}$ covers two to three orders of magnitude across the spectral range, with a minimum above strong vibrational resonances due to the destructive interference between the positive nonresonant electronic response and the negative vibrationally resonant response. This can be also clearly seen in the retrieved real part $\text{Re}(\bar{\chi })$, which crosses zero in these spectral regions. Accurately measuring the CARS intensity over a dynamic range of 3 orders of magnitude can be challenging and requires suited acquisition parameters and background subtraction.

Generally, the Im(χ¯) spectra resemble the Raman spectra shown in Figures 2–5 keeping in mind that the latter show *R*, which contains an additional *ω* ^−1^ factor to directly represent the sum rule.

Starting with water and heavy water (Figure 6), we evaluate the O–H and O–D stretch peak areas in Im(χ¯) over the ranges 2900–3700 cm^−1^ and 2100–2900 cm^−1^, respectively, using a linear baseline matching the end points, with the results given in Table 2. Because Im(χ¯) is scaling with the bond density in the focal volume, we evaluate the area per bond density *A*/*N*, using the bond density $N=DN_{b}/M$, with the molecular weight *M*, the mass density *D* and the number of bonds per molecule *N*_b_. Note that the theoretical expression for *A*/*N* is given in Equation (10), showing a proportionality to the vibrational wavenumber under isotope substitution. The ratio *ϱ* of the area per bond density between –H and –D bonds is found to be 1.57, and using a vibrational wavenumber ratio of 1.35, the sum rule factor is η=1.16, that is, the –H bonds have a 16% larger area than predicted by the sum rule.

**TABLE 2:** Im(χ¯) peak areas (*A*) and derived quantities for O–H, O–D, C–H and C–D associated peaks across all investigated molecules

Molecule	Bond	*N*_b_	*A*	*N*	*A*/*N*	*ϱ*	*η*
			(cm^−1^)	(nm^−3^)	(cm^−1^ nm^3^)		
H_2_O	O–H	2	528	6.67	79.2	1.57	1.16
D_2_O	O–D	2	336	6.67	50.4		
SA	C–H	4	167	3.18	52.5	1.70	1.26
D4‐SA	C–D	4	98	3.18	30.8		
OA	C–H	33	619	6.29	98.4	1.63	1.20
D17‐OA	C–H	16	300	3.05	98.4	1.63	1.20
	C–D	17	196	3.24	60.5
LA	C–H	31	512	5.99	85.5	1.47	1.09
D11‐LA	C–H	20	321	3.86	83.2	1.43	1.06
	C–D	11	123	2.12	58.0		

*Note*: *N* is the bond density, *A*/*N* is the area per bond density and *ϱ* is the ratio of *A*/*N* between –H and –D bonds. *η* is *ϱ* divided by the wavenumber ratio, which is predicted to be unity by Equation (10).

Moving to succinic acid (Figure 7), the spectrally sharp resonances lead to a strong modulation of $\text{Re}(\bar{\chi })$, reaching strongly negative values above resonance and a large maximum Im(χ¯) of around 4. We evaluate C–H and C–D stretch peak areas in Im(χ¯) over the ranges 2600–3250 cm^−1^ and 1950–2400 cm^−1^ using a linear baseline matching the end points. The results are again shown in Table 2, giving ϱ=1.70 and a sum rule factor of η=1.26.

OA and LA fatty acids are shown in Figures 8 and 9. The large number of C–H stretch vibrations in OA leads to a large resonant response, with values of $\text{Re}(\bar{\chi })$ spanning from −2 to +6, and Im(χ¯) reaching 5. The peak areas are evaluated as above and are given in Table 2, resulting in ϱ=1.63, both comparing the C–H in OA with the C–D in D17‐OA, and for the C–H in D17‐OA, corresponding to a sum rule factor of η=1.20. Notably, the area per bond density *A*/*N* is nearly twice that in SA, indicating some coherent coupling within the long C–H chains.

For LA, the results are similar to OA. Notably, the C=C peak is stronger, consistent with the observation in Raman. The peak areas are evaluated as above and are again given in Table 2, resulting in ϱ=1.47 comparing the C–H in LA with the C–D in D11‐LA, and ϱ=1.43 for the C–H in D11‐LA, corresponding to sum rule factors of η=1.09 and 1.06. This better agreement with the sum rule is mostly due to the reduction of the area per bond density *A*/*N* for the C–H bonds compared with OA.

Overall, the sum rule factors are similar to the ones observed in spontaneous Raman, see Table 1. We note that the accuracy of the retrieved areas is estimated to be better than 10%, limited by slightly different focusing for the reference spectrum taken in the coverslip glass, and the PCKK retrieval. However, we can see that the ratio *ϱ* seen in the deuterated compounds alone (D17‐OA and D11‐LA) and the ones seen comparing with separate measurements (OA and LA) show a maximum difference of 3%, indicating that systematics are in the few percentage range. The up to 20% higher area of the O–H and C–H vibrations than expected from the sum rule could be related to anharmonicity and coherent coupling effects.

## 5 CONCLUSIONS

In conclusion, we have shown that the Raman scattering coefficients and the coherent Raman susceptibility $\bar{\chi }$ of the –H stretch vibrations approximately scale with the vibrational frequency under isotopic substitution of hydrogen to deuterium. This is consistent with theoretical expectations of a sum rule for the Raman polarizability. We have reported quantitative $\bar{\chi }$ spectra for four relevant substances, water, succinic acid, oleic acid and linoleic acid, and their deuterated isoforms. Generally, –H stretch vibrations have a susceptibility higher by approximately 50% than –D stretch vibrations.

These results highlight that deuterium isotope substitution, albeit providing a simple way to increase chemical specificity in Raman and CRS methodologies by shifting vibrational resonances to the silent region, is accompanied by a signal reduction. Our finding will be relevant to the growing field of Raman and CRS imaging and sensing applications using deuterium isotope‐labelling, for example, to accurately estimate detection limits and quantitatively interpret the data.

## CONFLICT OF INTEREST

There are no conflicts to declare.

## Supporting information



JRS_6164_chi3CDCH_SM.pdf

## Data Availability

Information about the data created during this research, including how to access it, is available from Cardiff University data archive (http://doi.org/10.17035/d.2021.0132639812).
