# Supplementary material for: Quantification of the nonlinear susceptibility of the hydrogen and deuterium stretch vibration for biomolecules in coherent Raman micro‐spectroscopy
Source: J Raman Spectrosc. 2021 Jun 23;52(9):1540–51. doi: 10.1002/jrs.6164 (PMC9627839; doi:10.1002/jrs.6164)
Supplement: Supplementary file 1 — JRS_6164_chi3CDCH_SM.pdf [file JRS-52-1540-s001.pdf]

Supplementary Information - Quantification of the nonlinear susceptibility of the hydrogen and deuterium stretch vibration for biomolecules in coherent Raman micro-spectroscopy

Dale Boorman,[1] Iestyn Pope,[1] Francesco Masia,[1,2] Peter Watson,[1] Paola Borri,[1] and Wolfgang Langbein[2,*]

[1] *Cardiff University, School of Biosciences, Museum Avenue, Cardiff CF10 3AX, UK*

[2] *Cardiff University, School of Physics and Astronomy, The Parade, Cardiff CF24 3AA, UK*

S1. RAMAN CROSS-SECTION

The Raman cross-section equation Eq.(1) was derived from Eq.(3) in [1], given by

$$\frac{d\sigma_l}{d\Omega} = \frac{\pi^2}{90\epsilon_0^2} \frac{(\nu_0 - \nu_l)^4}{1 - \exp(-h\nu_l c/k_{\rm B}T)} S_l \,, \tag{S1}$$

by converting the wavenumbers to angular frequencies, and using known definitions of constants

$$\omega_l = 2\pi c\nu_l, \;\omega_0 = 2\pi c\nu_0, \;\hbar = h/(2\pi), \;c = 1/\sqrt{\epsilon_0\mu_0}$$

as follows

$$\frac{\pi^2}{90\epsilon_0^2}\left(\nu_0 - \nu_l\right)^4 = \frac{\pi^2}{90\epsilon_0^2(2\pi c)^4}\left(\omega_0 - \omega_l\right)^4 = \frac{\epsilon_0^2\mu_0^2}{90\epsilon_0^2 2^4\pi^2}\left(\omega_0 - \omega_l\right)^4 = \frac{\mu_0^2}{1440\pi^2}\left(\omega_0 - \omega_l\right)^4 \,.$$

Notably, Eq.(S1) is stated in [1] without derivation, with the reference [2] when discussing the polarizabilities. In [2] chapter 16, Raman scattering is described, and Eq. (16.6b) gives the Stokes intensity in a similar form, but not for the specific polarization and direction. Importantly, [1] reports quantitative comparisons between experiment and theory, so that Eq.(3) is likely correct. It should be noted that this expression assumes emission into vacuum (suited since the work considers gas phase Raman), while for emission into a medium of refractive index different from unity an according correction must be applied. However, in our work we are only concerned with the relative changes for isotope exchange for an unchanged refractive index.

We note that in [3], a work having the common author Montero with [1], the same expression is used, and the additional reference [4] is given. In [4] Eq.(2), the cross-section is given as

$$\frac{d\sigma_l}{d\Omega} = \frac{(2\pi)^4}{45} \frac{b_l^2\left(\nu_0 - \nu_l\right)^4}{1 - \exp(-h\nu_l c/k_{\rm B}T)} \left(45\alpha_l'^2 + 7\gamma_l'^2\right) \tag{S2}$$

with the zero-point motion amplitude

$$b_l = \sqrt{\frac{h}{8\pi^2\nu_l c}}$$

TABLE S1. Chemical composition and structure for the investigated deuterium-labelled and unlabelled molecules. Structures were generated using ChemSketch software.

Molecule	C-H / C-D	Structure
water H_2O		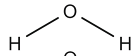
heavy water D_2O		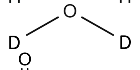
succinic acid $C_4H_6O_4$	4 / 0	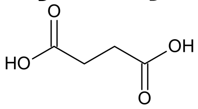
D4-succinic acid $C_4H_2D_4O_4$	0 / 4	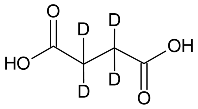
oleic acid $C_{18}H_{34}O_2$	33 / 0	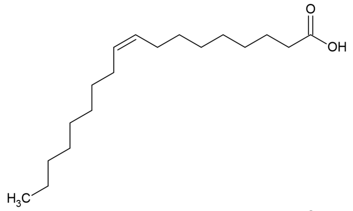
D17-oleic acid $C_{18}H_{17}D_{17}O_2$	16 / 17	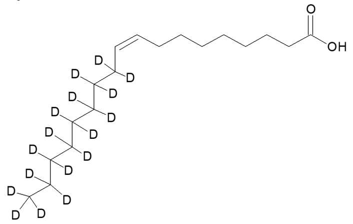
linoleic acid $C_{18}H_{32}O_2$	31 / 0	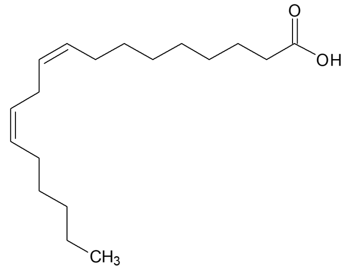
D11-linoleic acid $C_{18}H_{21}D_{11}O_2$	20 / 11	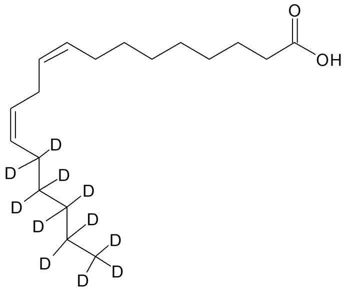

* langbeinww@cf.ac.uk

of the vibrational mode, and it is stated that they follow from relations given in [2]. Notably, here α'_l and γ'_l are the trace and anisotropy of the "derived polarizability tensor associated with the normal coordinate Q_l". The dimensionless normal coordinate q_l is related to the mass weighted normal coordinate Q_l by

$$Q_l = \sqrt{\frac{\hbar}{\omega_l}} q_l$$

Accordingly, α'_l and γ'_l are related to the derivatives versus the dimensionless normal coordinates, $\bar{\alpha}'_l$ and $\bar{\gamma}'_l$ used in S_l of Eq.(S1), by

$$\alpha'_l = \sqrt{\frac{\omega_l}{\hbar}} \bar{\alpha}'_l\,, \quad \gamma'_l = \sqrt{\frac{\omega_l}{\hbar}} \bar{\gamma}'_l\,.$$

The zero point motion and coordinate conversion provide a prefactor in Eq.(S2) of

$$\frac{(2\pi)^4}{45} \sqrt{\frac{h}{8\pi^2 \nu_l c}}^2 \sqrt{\frac{\omega_l}{\hbar}}^2 = \frac{(2\pi)^4}{45} \frac{\hbar}{4\pi \nu_l c} \frac{\omega_l}{\hbar} = \frac{(2\pi)^4}{45} \frac{\omega_l}{2\omega_l} = \frac{(2\pi)^4}{90}$$

so that Eq.(S2) can be written as

$$\frac{d\sigma_l}{d\Omega} = \frac{(2\pi)^4}{90} \frac{(\nu_0 - \nu_l)^4}{1 - \exp(-h\nu_l c/k_\mathrm{B} T)} S_l$$

Compared to Eq.(S1) we are missing a factor of $(4\pi\epsilon_0)^2$, evidencing that Eq.(S2) was written in Gauss units. This is relevant for the polarizabilies α, γ, which are a charge times length per electric field. Electric fields E and charges q are converting between the unit systems as

$$E^\mathrm{Gauss} = E^\mathrm{SI} \sqrt{4\pi\epsilon_0}, \; q^\mathrm{Gauss} = q^\mathrm{SI} / \sqrt{4\pi\epsilon_0}\,,$$

so that

$$\alpha^\mathrm{SI} = \alpha^\mathrm{Gauss} 4\pi\epsilon_0, \; \gamma^\mathrm{SI} = \gamma^\mathrm{Gauss} 4\pi\epsilon_0\,,$$

yielding $S_l^\mathrm{SI} = S_l^\mathrm{Gauss}(4\pi\epsilon_0)^2$ and therefore Eq.(S2) is equivalent to Eq.(S1).

S2. CRS SUSCEPTIBILITY RESONANT APPROXIMATION

We consider the resonance

$$\frac{1}{\omega_l^2 - \Delta^2 + 2i\Delta\gamma}\,.$$

We assume $|\Delta/\omega_l - 1| \ll 1$, as well as $\gamma/\omega_l \ll 1$. We denote $\delta = \Delta - \omega_l$ so that $|\delta/\omega_l| \ll 1$. We thus have

$$\begin{aligned} &\omega_l^2 - \Delta^2 + 2i\Delta\gamma = \\ &\omega_l^2 - \omega_l^2 - 2\delta\omega_l - \delta^2 + 2i(\omega_l + \delta)\gamma = \\ &\omega_l \left(\delta\left(-2 - \delta/\omega_l\right) + 2i(1 + \delta/\omega_l)\gamma\right) \approx \\ &2\omega_l \left(-\delta + i\gamma\right) = \\ &2\omega_l \left(\omega_l - \Delta + i\gamma\right) \end{aligned}$$

where the approximation neglected δ/ω_l. As a result we find

$$\frac{1}{\omega_l^2 - \Delta^2 + 2i\Delta\gamma} \approx \frac{1}{2\omega_l} \left(\omega_l - \Delta + i\gamma\right)^{-1}$$

as used in Eq.(7). An equivalent derivation holds for the resonance at negative frequencies Δ for which we have $|\Delta/\omega_l + 1| \ll 1$.

[1] J. Martín and S. Montero, *J. Chem. Phys.*, 1984, **80**, 4610–4619.

[2] G. Placzek, in *Handbuch der Radiologie*, ed. E. Marx, Akademische Verlagsgesellshaft VI, 1934, ch. Die Rayleigh und Raman Streuung, pp. 209–374.

[3] J. M. Fernández-Sánchez and S. Montero, *J. Chem. Phys.*, 1989, **90**, 2909–2914.

[4] W. F. Murphy, W. Holzer and H. J. Bernstein, *Appl. Spectrosc.*, 1969, **23**, 211–218.
